# Need for gender-neutral human papillomavirus vaccination: Lessons from a retrospective epidemiologic investigation of human papillomavirus infections among outpatients presenting to a tertiary dermatology hospital in Central China from 2017 to 2024

**DOI:** 10.1016/j.ijregi.2026.100884

**Published:** 2026-03-25

**Authors:** Xianfeng Zhou, Han Mo, Zhijun Zhou, Yi Xu, Changxia Li, Xiaohua Tao

**Affiliations:** 1 Jiangxi Provincial Clinical Research Center for Skin Diseases, Branch of National Clinical Research Center for Skin Diseases, Dermatology Hospital of Jiangxi Province, Nanchang, China; 2 Evidence- Based Medicine Research Center, Jiangxi University of Chinese Medicine, Nanchang, China; 3 Jiangxi Engineering Research Center for Translational Cancer Technology, Jiangxi University of Chinese Medicine, Nanchang, China; 4 JXHC Key Laboratory of Skin Infection and Immunity, The Affiliated Dermatology Hospital of Nanchang University, Nanchang, China

**Keywords:** Human papillomavirus, Dermatology, Outpatient individuals, Co-infection, Epidemiology, Clinical presentations

## Abstract

**Objectives:**

This study aimed to analyze human papillomavirus (HPV) prevalence, genotype distribution, and associated clinical characteristics among outpatient individuals attending a tertiary dermatology hospital in Central China, to inform public health strategies.

**Methods:**

A retrospective analysis was conducted on electronic health records of 3001 outpatients from 2017 to 2024. HPV genotyping was performed using a flow fluorescent hybridization assay detecting 27 genotypes (17 high-risk [HR] and 10 low-risk [LR] types).

**Results:**

The overall HPV positivity rate was 31.4%, significantly higher in males than females (34.8% vs 28.9%, *P* <0.001). The predominant HR-HPV genotypes were HPV16 (20.9%), HPV52 (20.1%), and HPV51 (13.8%); LR-HPV infections were mainly HPV6 (51.1%), HPV11 (22.8%), and HPV43 (14.4%). Multi-genotype infections occurred in 39.6% of cases, notably, among young, single individuals. Genital warts were the primary clinical manifestation. Asymptomatic presentation was significantly more prevalent in HR-HPV vs LR-HPV infections (*P* <0.001). Cases were geographically concentrated near the hospital, indicating health care access disparities.

**Conclusion:**

The findings highlight high HPV prevalence and significant co-infection rates in Central China, emphasizing the risk of hidden HR-HPV transmission. Implementing gender-neutral vaccination (covering prevalent genotypes including 6, 11, 16, 18, 52, and 58) and enhancing screening (e.g. colposcopy, self-sampling in rural areas) are crucial public health priorities.

## Highlights

• Genital warts were predominantly observed in male and female cases.
• Positive rate of human papillomavirus (HPV) was significantly higher in males than in females.
• Multi-genotype infections were observed in 39.6% of cases, particularly, among young, single individuals.
• High proportion of asymptomatic high-risk HPV infections poses challenge for early intervention.
• HPV6 was the most dominant genotype, with 46.2% co-infected cases observed in the cohort.

## Graphical abstract

**Figure:**
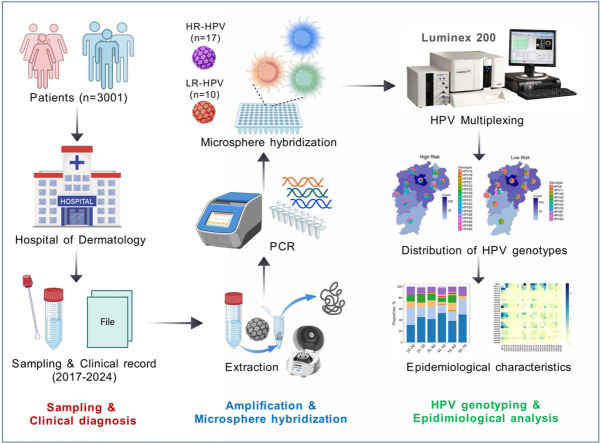

## Introduction

Human papillomavirus (HPV) poses a significant public health threat given its well-established role in the development of cervical cancer [1]. HPV infections are among the most prevalent viral infections in humans, contributing to one in 20 new cancer cases and exerting a particularly high burden on women [[2], [3], [4]]. In addition to its recognized association with cervical cancer, HPV has been linked to a wide spectrum of diseases, including various forms of skin lesions such as warts, flat warts, epidermodysplasia verruciformis, and precancerous lesions [5].

HPV, a member of the Papillomaviridae family, is a non-enveloped, double-stranded DNA virus with a circular genome of approximately 8000 base pairs [6]. At present, over 200 identified genotypes of HPV have been categorized into high-risk (HR) and low-risk (LR) types based on their oncogenic potential [7,8]. Among the approximately 200 HPV genotypes, around 40 infect the anogenital tract, a fact that has led to public health concerns regarding the sexual transmission of HPV [9]. HPV genotypes that are classified as HR are known to be strongly associated with malignancies, including cervical, anal, and oropharyngeal cancers [3,10]. HPV16 and HPV18 are the most prevalent HR-HPVs, accounting for >70% of cervical cancer cases worldwide, and additional HR-HPVs include HPV31, HPV35, HPV45, HPV51, and HPV56, which cumulatively contribute to 20-25% of cervical cancer [11]. Recent epidemiologic studies have identified a rise in the prevalence of other HR-HPVs, such as HPV52, HPV58, and HPV33, particularly, among Asian populations [[12], [13], [14], [15]]. LR-HPVs predominantly manifest as benign lesions, including genital warts (e.g. HPV6 and HPV11) and low-grade squamous intraepithelial lesions [16]. Although non-oncogenic, LR-HPV infections impose a significant clinical burden due to their high transmissibility and recurrence rates [3]. The prevalence and distribution of HPV genotypes have been a significant focus in recent decades in high-income and low-income countries [4,9,15,[17], [18], [19], [20]]. Recent studies have identified multi-infection patterns and the co-infection preference of HPV types among gynecological outpatients in China, underscoring the necessity of screening for HR-HPV and LR-HPV in younger women and detection in older women [21]. Multi-infection has been identified as a prevalent phenomenon, with the potential to exacerbate disease progression [22]. Seong *et al.* found that persistent HPV-16/58 infections in Korean women led to enhanced cervical intraepithelial neoplasia progression [14].

Dermatology and venereology clinics represent a crucial gateway for HPV screening, thereby enhancing the likelihood of timely screening and diagnosis of HPV [23,24]. The present study is a retrospective analysis of the HPV prevalence and distribution among outpatient individuals presenting to the Department of Dermatology and Venereology at a tertiary hospital in Central China from 2017 to 2024. The results revealed that the top three prevalent HR-HPV and LR-HPV genotypes, irrespective of gender, were HPV16, 52, and 51 and HPV6, 11, 43, respectively. A statistically significant disparity was observed in the positive rate of HPV between males and females, with a *P*-value less than 0.001. Moreover, 39.6% cases were infected by multiple genotypes (2-7 genotypes), and the single/unmarried population had significantly higher risk of multi-infection of HPV than the married population did (*P* = 0.018). The findings of this study underscore the need for the promotion of multi-valent HPV vaccination not only for females but also for males in the future. Furthermore, the promotion of ease-to-use and low-cost HPV self-sampling kits will expedite the process of early screening HR populations in suburban and rural areas, in which access to professional dermatology and venereology clinics is very limited.

## Materials and methods

### Study population

Data were obtained from the electronic health records of 3001 patients who sought medical services and underwent HPV testing at the dermatology department of a tertiary dermatology hospital in Jiangxi, China, during 2017-2024. Data on clinical characteristics, including gender, age, diagnosis, and symptoms found during routine examinations, were collected by medical specialists. Epidemiologic characteristics, risk factors for HPV infection, and clinical presentation of individuals positive for HPV were systematically collected and characterized according to the pipeline (Supplementary Figure 1). The retrospective study was approved by the Institutional Review Board (IRB) (approval number KY2025-16-01). Due to the retrospective nature of the study and the use of de-identified patient data, informed consent was waived by the IRB. The study was conducted in accordance with the ethical standards of the Declaration of Helsinki and the subsequent amendments.

### Specimen collection and processing

The following sample types were collected for analysis: cervical, female urethral, and male urethral secretions were collected for further analysis. (a) Cervical samples: a cervical brush was rotated 4-6 times at the cervical to collect epithelial cells. Subsequently, the brush head was placed in a preservation tube, and the handle was snapped off. (b) Female urethral samples: after the cleansing process with saline, a swab was inserted 2 cm into the urethra and preserved. (c) Before specimen collection, participants were required to abstain from vaginal medications and intercourse for a period of 24-72 hours. In addition, to ensure adequate specimen quality, participants were asked to hold their urine for at least 1 hour before the urethral swab procedure. Furthermore, menstruating women were sampled 10-18 days post-menstruation.

### Laboratory testing and genotyping of HPV

This study aimed to use a flow fluorescent hybridization assay with a commercial Human Papillomavirus Genotyping Kit (Shanghai Tellgen Life Science Co., Ltd.) to qualitatively detect and genotype HPV from clinical specimens. The kit was used to identify 27 genotypes, including 17 HR-HPV genotypes (HPV16, 18, 26, 31, 33, 35, 39, 45, 51, 52, 53, 56, 58, 59, 66, 68, and 82) and 10 LR-HPV genotypes (HPV6, 11, 40, 42, 43, 44, 55, 61, 81, and 83). The protocol entailed the extraction of nucleic acids, followed by polymerase chain reaction (PCR) amplification, hybridization, and analysis via a multiplex detection system. Specifically, samples were vortexed and treated with extraction buffer (100°C, 10 minutes). After a centrifugation process at 2000 revolutions per minute for a duration of 3 minutes, the upper layer of the mixture was collected and retained. Subsequently, the PCR reaction mixture, comprising 5 µL of primer mix and 10 µL of PCR master mix, 0.8 µL of Taq polymerase, and 5 µL of template DNA, was amalgamated and subjected to the following amplification conditions: initial denaturation at 95°C for 3 minutes; five cycles of 95°C for 30 seconds, 58°C for 30 seconds, and 72°C for 30 seconds; 35 cycles of 95°C for 30 seconds, 55°C for 30 seconds, and 72°C for 30 seconds; and final extension at 72°C for 3 minutes. Subsequently, the PCR products (3 µL) were combined with the microsphere hybridization solution (22 µL) at 48°C for 30 minutes. Thereafter, 75 µL of streptavidin-phycoerythrin was added, and the resulting fluorescence signals were analyzed using the Luminex 200 Flow Analyzer (Luminex Corporation). Negative (non-HPV sequence) and positive (globin gene) controls were incorporated as internal controls.

### Statistical analysis

R Studio (version 4.4.3) was used in conjunction with packages such as ggplot2, scatterpie, sf, etc. to produce geographical plots and alluvial plots. Similarly, Python (version 3.12.4) was used in conjunction with the matplotlib and seaborn libraries to produce heat maps and other visualizations. Comparisons between groups were made using the Pearson chi-square test, with a statistical significance of *P* <0.05.

## Results

### General information of the study population

**Figure 1:**
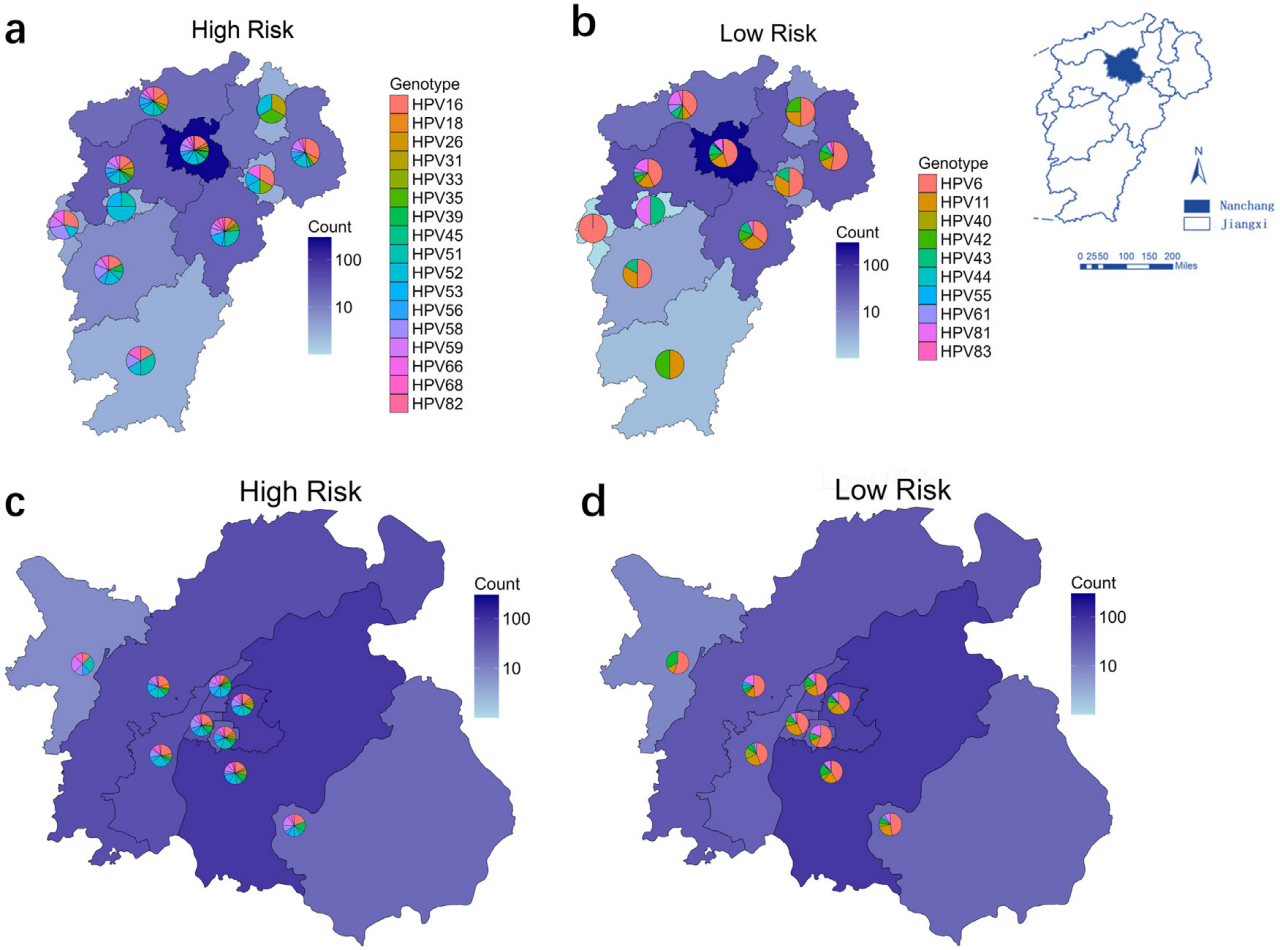
Characterization of the geographic distribution of HPV genotypes in Jiangxi, China. (a) High-risk-HPV genotype proportion in different cities of Jiangxi; (b) low-risk-HPV genotype proportion in different cities of Jiangxi; (c) high-risk-HPV genotype proportion in each district of the capital city of Jiangxi; (d) low-risk-HPV genotype proportion in each district of the capital city of Jiangxi. HPV, human papillomavirus.

A total of 3001 individuals aged 3-84 years received HPV detection during the study period (2017-2024). The overall positivity rate of HPV was 31.4%, and the positive rate in males (34.8%, n = 1238) was significantly higher than that in females (28.9%, n = 1763) (*P* <0.001), as shown in Supplementary Table 1. Among the identified individuals, the population aged 21-50 years constituted the predominant demographic, accounting for 79.0% of the sample. The remaining 21.0% of the sample consisted of individuals below the age of 20 years or above the age of 50 years. Furthermore, a statistically significant variation in HPV positivity rates was observed across different age groups (*P* <0.001). An analysis of the living addresses indicated a provincial-level geographical distribution of HR-HPV and LR-HPV cases, as displayed in Figures 1a-1b. The genotype diversity of different cities was found to be associated with the number of HPV cases. It was found that the proportion of HPV16 was significantly high in the eastern region of Jiangxi (Figure 1a). The majority of cases (69.3%) were from outpatient individuals living in Nanchang city, the capital of Jiangxi Province and the location of the hospital. It was found that the HR-HPV or LR-HPV genotype composition in each district of Nanchang shared a similar pattern (Figures 1c-1d). Geographically, the number of patients and HPV cases exhibited a positive correlation with distance from their living addresses to the hospital. This finding underscores the importance of accessibility to dermatology clinics in the detection and early diagnosis of HPV.

### Epidemiologic characteristics of HPV–positive cases

**Figure 2:**
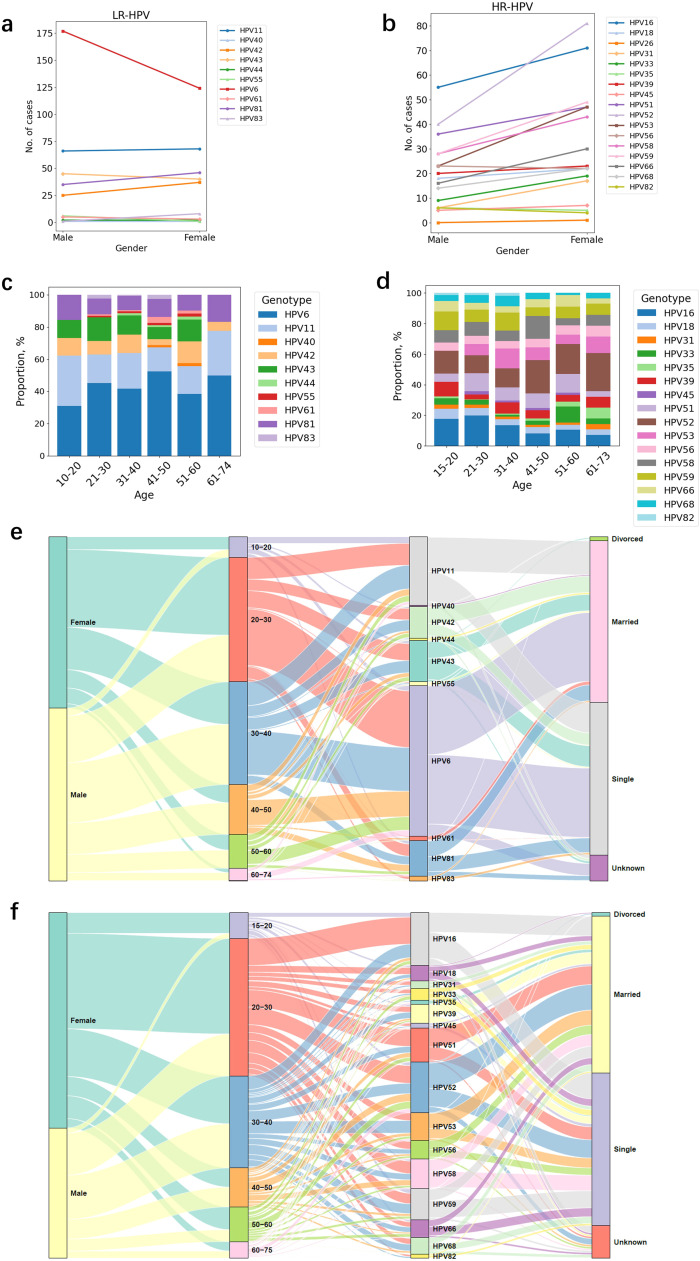
Gender, age, and marital characteristics of HPV infection. (a) Number of each LR-HPV genotypes in males and females; (b) number of each HR-HPV genotypes in males and females; (c) LR-HPV genotype proportion in different age groups; (d) HR-HPV genotype proportion in different age groups; (e) demographic characteristics of individuals infected with LR-HPV; (f) demographic characteristics of individuals infected with HR-HPV. HR, high-risk; HPV, human papillomavirus; LR, low-risk.

**Figure 3:**
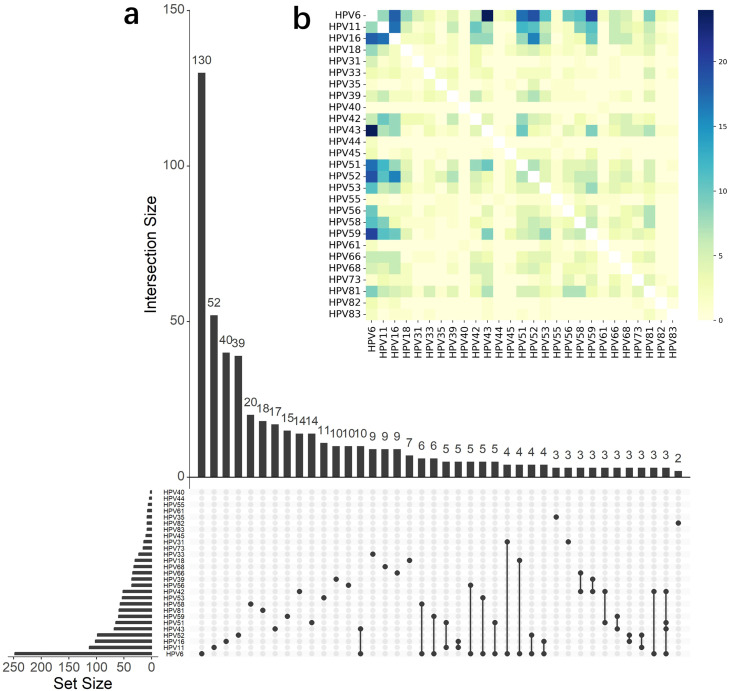
Interactive analysis of co-infection of HPV genotypes. (a) UpSet analysis of each genotype; (b) heatmap of genotype–genotype co-infections. HPV, human papillomavirus.

Among the individuals positive for HPV, 568 were infected by a single genotype, whereas 373 were infected by multiple genotypes. The mean age of HPV cases is 36.1 (95% confidence interval: 34.9-37.3) and 32.9 (95% confidence interval: 31.9-35.2) years in males and females, respectively. The mean age of the HPV11-infected population was found to be the lowest, whereas the mean age of the HPV68-infected population was the highest among the studied populations. HPV6 was the predominant genotype across all age groups, accounting for a significant percentage (32.0%, n = 941) of LR-HPV–positive cases, followed by HPV11 (14.2%) and HPV43 (9.0%) (Figure 2a). In the case of patients positive for HPV, HPV 52, 16, and 51 were the three most prevalent genotypes among male and female patients (Figure 2b). As illustrated in Figure 3c, the genotype diversity of LR-HPV is higher in patients aged 30-60 years than in those younger than 30 years or older than 60 years. This pattern was not observed in HR-HPV cases, among whom similar genotype composition was observed in each age group (Figure 2d). However, it was observed that HPV53 was not detected in patients under 20 years of age, and HPV83 was not detected in patients over 40 years of age (Figure 2c). A statistically significant discrepancy was not observed between male and female subjects regarding mono-infection or multiple infections. Nevertheless, the incidence of mono-infections in patients between the ages of 30 and 50 years was found to be considerably higher than that observed in other age groups (*P* = 0.018).

Among the 941 HPV–positive cases, 373 (39.6%) had multi-infections with two to seven genotypes (Supplementary Figure 2a). HPV6 was the most dominant genotype, with 46.2% (n = 139) co-infected cases observed in this cohort (Supplementary Figure 2b). The single/unmarried population had significantly higher risk of multi-infection of HPV than the married population did (*P* = 0.018) (Table 1). However, there is no significant difference of multi-infection ratio between males and females (*P* = 0.805), indicating that health education and gender-neutral vaccination promotion targeting the young, single population are necessary. By analyzing the co-infection mode, an UpSet analysis and heatmap demonstrated that co-infections frequently occurred among HPV6, 11, 18, 42, 43, 51, 52, 53, 56, 58, 59, and 81 (Figure 3), in which six genotypes were covered by the available nine-valent HPV vaccines.

**Table 1:** Comparison of mono-infection and multi-infection of human papillomavirus in terms of gender, age, marital status, and symptom.

Item	Mono-infection	Multi-infection	χ²-Value	*P*-Value
**Gender**			0.06075	0.805
Female	306	204		
Male	262	169		
**Age group, years**			20.6039	<0.001
10-20	23	27		
21-30	189	148		
31-40	197	92		
41-50	88	42		
51-60	52	46		
>60	19	18		
**Marital status**			10.013	0.018
Married	221	142		
Single	164	139		
Unknown	53	21		
**Symptom**			16.752	0.033
Asymptomatic	178	98		
Abnormal discharge	17	23		
Genital papules	56	51		
Genital warts	132	105		
Other genital symptoms	19	9		
Perianal warts	31	18		
Perianal papules	11	15		
Others	33	20		

### Clinical characteristics

The clinical diagnosis information was then extracted, and the clinical symptoms were collected accordingly. The clinical records indicated that the mono-infection of HPV exhibited a notably higher proportion of asymptomatic cases than cases of multi-infections (*P* = 0.033). Excluding cases that were asymptomatic, genital warts were the most common presentation, followed by genital papules. The clinical presentation also encompassed perianal warts, perianal papules, abnormal discharge, and other genital symptoms. Regardless of mono- or multi-infection, the most common clinical symptoms were more frequently diagnosed in cases of LR-HPV infections, whereas the proportion of asymptomatic presentations in cases of HR-HPV infections was significantly higher than that in LR-HPV cases (*P* <0.001) (Table 2). This result suggests that there was a potentially higher risk of insidious transmission of HR-HPV. Furthermore, the proportion of HR-HPV in female subjects was found to be significantly higher than in male subjects (54.8% vs 43.3%, *P* <0.001) (Table 2). To explore the genotype-specific connection with clinical symptoms, Sankey diagrams were constructed, and the observation indicated that HPV6 and HPV11 contributed to the highest proportion of genital warts, genital papules, perianal warts, and papules (Supplementary Figure 3a). However, a lack of statistical significance was observed between clinical symptoms and HR-HPV genotypes (Supplementary Figure 3b). In light of these findings, the potential benefits of incorporating colposcopy or anoscopy into the early cancer screening protocol for individuals who are HR-HPV–positive warrant further consideration.

**Table 2:** Comparison of HR-PHV and LR-HPV infections in terms of gender, age, marital status, and symptom.

Item	HR-HPV	LR-HPV	χ²-Value	*P*-Value
**Gender**			12.430	<0.001
Female	292	241		
Male	181	237		
**Age group, years**			3.443	0.752
18-20	21	22		
21-30	179	169		
31-40	129	149		
41-50	62	67		
51-60	54	47		
61-80	17	17		
<18	11	7		
**Marital status**			1.173	0.760
Married	227	224		
Single	193	208		
Unknown	48	41		
**Symptom**			30.908	<0.001
Asymptomatic	192	141		
Genital warts	129	186		
Other genital symptoms	19	14		
Genital papules	60	86		
Perianal papules	16	22		
Perianal warts	25	35		
Abnormal discharge	28	25		
Others	41	23		

HR, high-risk; HPV, human papillomavirus; LR, low-risk.

## Discussion

This study investigated the epidemiologic and clinical characteristics of HPV infections among outpatient individuals from a tertiary hospital of dermatology in Central China, highlighting significant disparities in HPV prevalence, genotype distribution, and infection patterns between genders and age groups. The findings of this study emphasize the importance of developing public health strategies that are specifically tailored to the needs of the population in this region. The objective of these strategies is to reduce the transmission of HPV and the subsequent disease burden that is associated with it.

A salient finding of this study is the markedly elevated HPV positivity rate in males (34.8%) than in females (28.9%) (*P* <0.001). This observation is consistent with the findings of recent studies that have indicated an increase in HPV prevalence among males, particularly, in regions where male-targeted vaccination programs are limited [4,7]. The elevated rate among males may be indicative of disparities in sexual behavior, immune response, or health care–seeking patterns because males are less inclined to undergo routine HPV screening than females. These findings underscore the pressing need to extend HPV vaccination to include males, as recommended by the nine-valent HPV vaccine guidelines, with the objective of reducing transmission and preventing HPV-related cancers in both genders [1].

It is noteworthy that co-infections involving multiple HPV genotypes constituted ∼40% of the cases, with young and unmarried individuals demonstrating a heightened susceptibility to multi-infections. This finding is consistent with global trends, where multi-infections are increasingly recognized as a driver of persistent HPV infection and disease progression [2,3]. The predominance of HPV6, 11, 16, 52, and V51 in HR-HPV and LR-HPV categories underscores the importance of multi-valent vaccines, which cover these genotypes [15,25]. However, the data demonstrate that only five of the prevalent co-infected genotypes (HPV6, 11, 16, 18, and 52) are currently incorporated into nine-valent vaccines, indicating a potential lacuna in coverage for other prevalent genotypes, including HPV51 and 58. The findings presented herein support the development of region-specific vaccine formulations and the expansion of genotype coverage to address local epidemiologic patterns.

The high proportion of asymptomatic HR-HPV infections (*P* <0.001 vs LR-HPV) poses a significant challenge for early detection and intervention. Asymptomatic carriers may unknowingly transmit HR genotypes, thereby contributing to insidious cancer development. This underscores the significance of incorporating colposcopy or anoscopy into screening protocols for individuals positive for HR-HPV, particularly, in resource-constrained settings where advanced diagnostic instruments are limited [5]. Furthermore, the implementation of self-sampling kits, as outlined in this study, has the potential to augment screening accessibility within rural and suburban populations, thereby aligning with global initiatives to decentralize HPV testing [9,26].

A geographical analysis revealed that the majority of cases originated from urban areas in close proximity to the tertiary hospital, thereby highlighting significant disparities in health care access. This finding aligns with the observations made in other low- and middle-income regions, where a strong correlation has been identified between HPV detection rates and proximity to specialized clinics [27]. To rectify this inequity, it is imperative that mobile screening units and community-based education programs be given priority to reach underserved populations.

This study is not without its limitations. First, the retrospective design may introduce selection bias because the data were limited to individuals seeking care at a single tertiary center. Second, the absence of longitudinal data impedes the evaluation of infection persistence or its progression to malignancy. Subsequent longitudinal studies that encompass viral load quantification and genomic sequencing have the potential to elucidate transmission dynamics and oncogenic mechanisms [28]. Finally, the absence of self-reported sexual behavior data constrained the investigation of behavioral risk factors. In conclusion, the findings of this study advocate for the implementation of gender-neutral HPV vaccination, the enhancement of multi-valent vaccine coverage, and the decentralization of screening strategies in Central China. Addressing these priorities will necessitate collaborative efforts between public health authorities, clinicians, and policymakers to reduce the growing burden of HPV-associated diseases.

## Declaration of competing interest

The authors have no competing interests to declare.

## Appendix Supplementary materials

Image, application 1
