# Supplementary material for: Need for gender-neutral human papillomavirus vaccination: Lessons from a retrospective epidemiologic investigation of human papillomavirus infections among outpatients presenting to a tertiary dermatology hospital in Central China from 2017 to 2024
Source: IJID Reg. 2026 Mar 25;19:100884. doi: 10.1016/j.ijregi.2026.100884 (PMC13092576; doi:10.1016/j.ijregi.2026.100884)
Supplement: Supplementary file 1 [file mmc1.docx]

**Supplementary Figure 1. Flowchart of genotyping, epidemiological and clinical analyses of HPV in outpatient individuals.** Segmented data collection and analysis was adopted based on the integrity of the data.


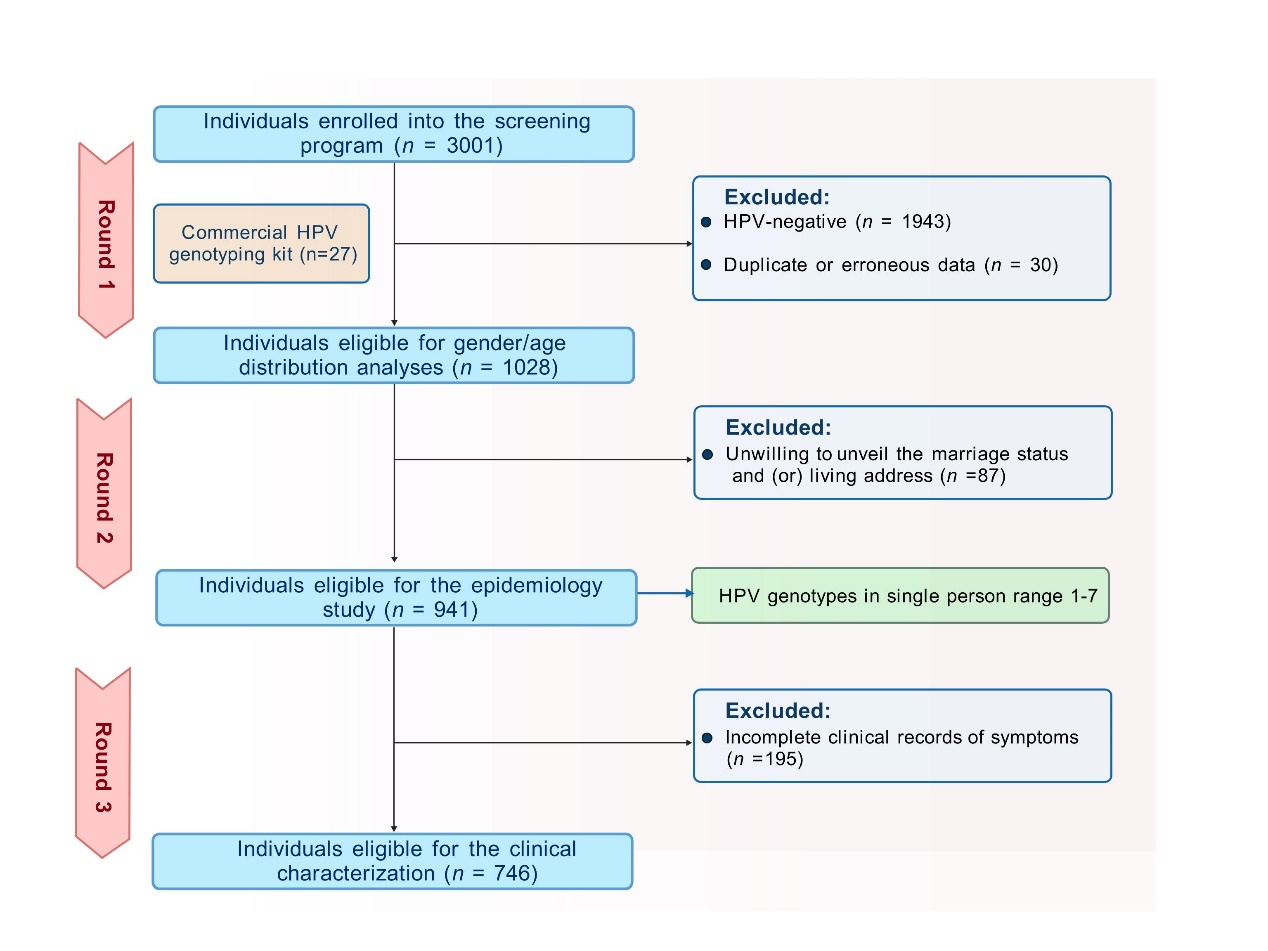


**Supplementary Figure 2. Number of cases of each genotype in mono-infected or multi-infected individuals.**


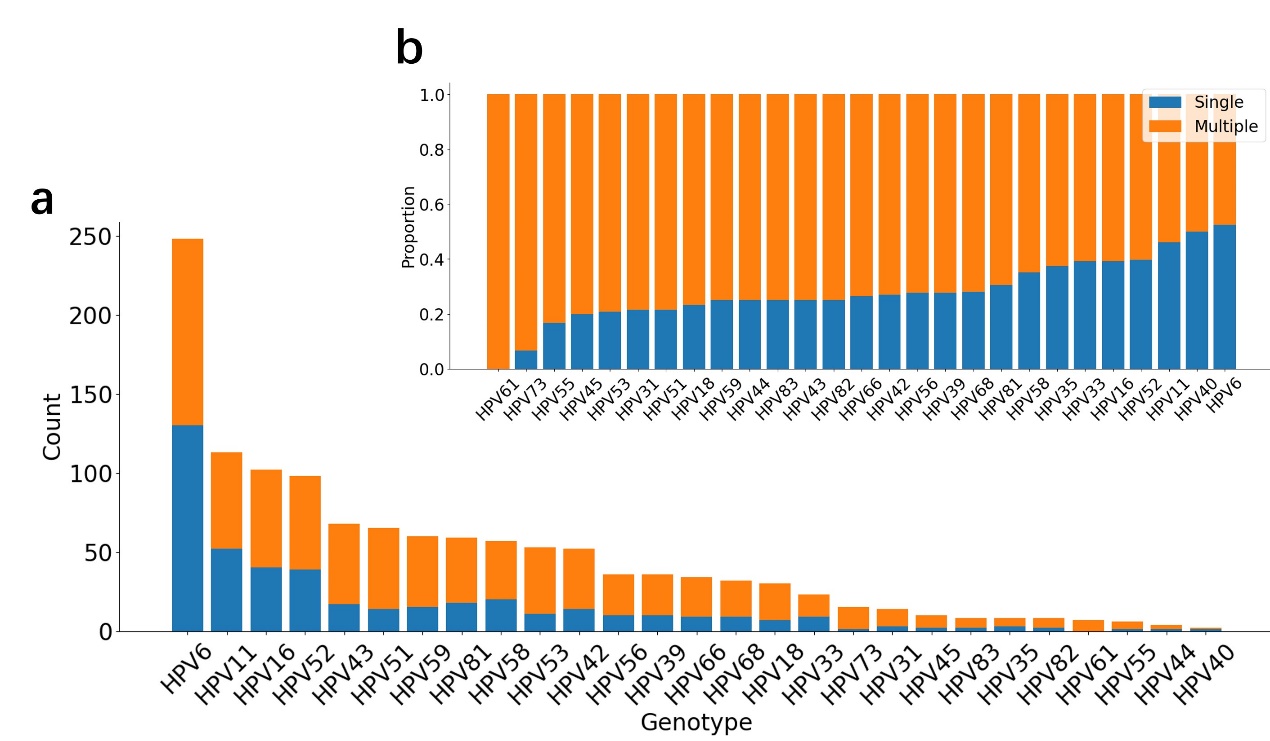


**Supplementary Figure 3. Clinical characteristics of each genotype in different age groups.** a. Clinical characteristics of LR-HPV infections; b. Clinical characteristics of HR-HPV infections.


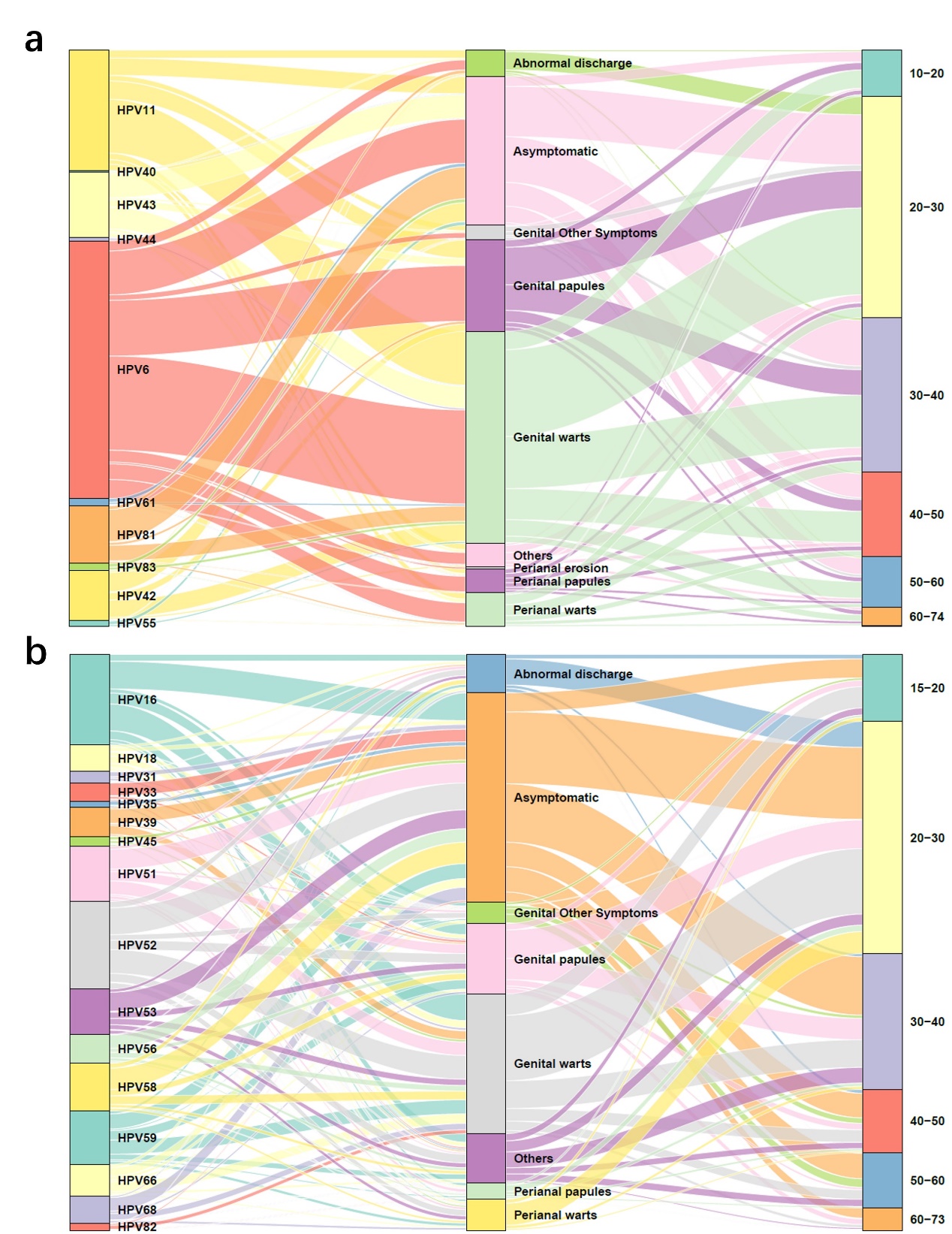


**Supplementary Table 1** Demographic characteristics of HPV-positive and HPV-negative individuals

| **Iterm** | **HPV(+)** | **HPV(-)** | **χ²-value** | **P-value** |
| --- | --- | --- | --- | --- |
| **Gender** |  |  | 11.436 | <0.001 |
| Male | 431 | 807 |  |  |
| Female | 510 | 1253 |  |  |
| **Age group** |  |  | 27.414 | <0.001 |
| 3-20 | 74 | 122 |  |  |
| 21-30 | 350 | 656 |  |  |
| 31-40 | 270 | 702 |  |  |
| 41-50 | 124 | 357 |  |  |
| 51-60 | 88 | 174 |  |  |
| >60 | 35 | 48 |  |  |
